# Changing paradigms in pediatric cancer care – the contemporary landscape and perspectives for India

**DOI:** 10.3332/ecancer.2025.1931

**Published:** 2025-06-24

**Authors:** Badira Cheriyalinkal Parambil, Nirmalya Roy Moulik, Venkata Rama Mohan Gollamudi, Shyam Srinivasan, Chetan Dhamne, Akanksha Chichra, Gaurav Narula, Mukta Ramadwar, Sumeet Gujral, Tanuja Shet, Epari Sridhar, Poonam Panjwani, Uma Sakhadeo, Siddhartha Laskar, Nehal Khanna, Jifmi Jose Manjali, Sajid Qureshi, Vasundhara Patil, Akshay Baheti, Sneha Shah, Kunal Gala, Pappagudi Subramanian, Prashant Tembhare, Nikhil Patkar, Gaurav Chatterjee, Sweta Rajpal, Dhanlaxmi Shetty, Maya Prasad, Girish Chinnaswamy

**Affiliations:** 1 Division of Pediatric Oncology, Tata Memorial Hospital, Homi Bhabha National Institute (HBNI), Mumbai 400012, India; 2 Department of Pathology, Tata Memorial Hospital, Homi Bhabha National Institute (HBNI), Mumbai 400012, India; 3 Department of Radiation Oncology, Tata Memorial Hospital, Homi Bhabha National Institute (HBNI), Mumbai 400012, India; 4 Department of Surgical Oncology, Tata Memorial Hospital, Homi Bhabha National Institute (HBNI), Mumbai 400012, India; 5 Department of Radiodiagnosis, Tata Memorial Hospital, Homi Bhabha National Institute (HBNI), Mumbai 400012, India; 6 Department of Nuclear Medicine, Tata Memorial Hospital, Homi Bhabha National Institute (HBNI), Mumbai 400012, India; 7 Department of Hematopathology, Tata Memorial Hospital, Homi Bhabha National Institute (HBNI), Mumbai 400012, India; 8 Department of Cytogenetics, Tata Memorial Hospital, Homi Bhabha National Institute (HBNI), Mumbai 400012, India

**Keywords:** pediatric cancer, cancer predisposition, immunotherapy, precision medicine, survivorship, perspectives

## Abstract

Advances in the diagnosis and management of childhood cancers have significantly improved survival, and 80% of those who have access to contemporary treatment are expected to survive into adulthood. Multimodality protocols incorporating high-intensity cytotoxic chemotherapy and radiotherapy may be associated with increased acute and delayed adverse effects, thereby compromising the quality of life. Furthermore, curative therapeutic options remain limited in the context of metastatic, relapsed or refractory disease as well as rare tumour entities. This has prompted a paradigm shift in pediatric oncology care in the contemporary era, encompassing multiple domains including cancer predisposition, immunotherapy, precision medicine and survivorship, aimed at optimising survival while minimising treatment-related toxicity and improving quality of life. While these advances are increasingly evident in high-income countries, several hurdles and challenges exist in the implementation of these strategies in low-income and middle-income countries (LMICs). Key barriers include restricted accessibility and affordability of newer and advanced diagnostic modalities and therapeutic agents, deficient infrastructure, non-availability of targeted agents and newer immunotherapy drugs, logistical and regulatory hurdles, limited access to clinical trials and inadequate long-term follow-up. Substantial changes are requisite to facilitate the translation of these changing paradigms into reality in India and LMICs.

## Introduction

Childhood cancer, although rare and constituting only around 3%–4% of all cancer diagnoses, has increased in incidence over the past several decades [1]. Dramatic advances in diagnosis and curative treatment strategies in pediatric malignancies have significantly improved the survival in this subset, and 80% who have access to contemporary treatment protocols are expected to survive into adulthood [2]. However, survival remains dismal with limited treatment options for various metastatic, relapse/refractory diseases and many rare tumours. Additionally, intense multimodality therapies incorporating cytotoxic chemotherapy have contributed to the high burden of late morbidity, impairing the quality of life [3]. This has led to a paradigm shift in pediatric oncology care, with risk-stratified, response-adapted treatment protocols replacing blanket treatment approaches. There has been increasing use of non-chemotherapy systemic treatments, which include immunotherapy and targeted therapy, genomics-focused pediatric precision oncology programs and establishment of varied models of survivorship programs, all of which focus on optimising cure with minimal morbidity and mortality.

The stark disparities in the incidence and patterns of childhood cancer between high-income countries (HICs) and low-income and middle-income countries (LMICs) extend to survival outcomes, with 80% of children with cancer cured of disease in HICs compared to 20% in LMICs [4, 5]. This article reviews the major shifts in pediatric oncology care and current perspectives, especially for India and applicable for other LMICs, in the fields of cancer predisposition syndromes, precision medicine, immunotherapy and survivorship.

## Cancer predisposition syndromes

Rapidly evolving research in the field of genetic susceptibility has found several germline mutations in predisposing genes in childhood cancer. Knowledge of the specific germline mutations facilitates the understanding of tumourigenesis, and helps direct patient care and genetic counselling. Approximately 10% of children with cancer are affected by monogenic cancer predisposition syndromes (CPSs) [6, 7]. CPS working groups have been established worldwide to provide consensus recommendations for the purposes of clinical, diagnostic and surveillance programs for carriers [8, 9].

### Cancer predisposition syndromes: current evidence

A landmark study by Zhang *et al* [7] utilised next generation sequencing (NGS) of 565 genes to identify germline mutations in 8.5% of 1,120 children and adolescents with diverse cancers [7]. Only 40% had a family history of cancer, of which only half had a cancer consistent with a known CPS [7]. Another study on the landscape of genomic alterations across childhood cancers showed that a pathogenic germline variant in one of the 162 genes tested was identified in 7.6% of the samples [6]. Some of the studies reporting on the genomic landscape in childhood cancer were restricted to certain tumour types and subtypes and may not be applicable to all childhood cancer cohorts [6, 7].

The varied approaches to diagnosis range from phenotype-driven, with candidate selection based on patient, tumour and physical characteristics, to a universal phenotype-agnostic comprehensive germline genetic testing or a combinatorial approach of both [10]. Each has its own advantages, with the phenotype-driven approach being clinically driven, thus creating an optimal balance of resource allocation, lower chance of ambiguous findings and identifying CPS in diseases associated with epigenetic changes or low-level mosaicism. Comprehensive genetic testing in all is best employed in research settings currently to understand the prevalence and spectrum of CPS [10].

The diagnostic and therapeutic implications of a diagnosis of CPS, though limited at present, could be significant in certain settings. In *DICER1* syndrome, which is associated with multiple rare tumours, the use of a germline gene panel can facilitate early diagnosis. Studies have demonstrated the utility of immune checkpoint inhibitors (ICIs) in germline-mismatch repair deficiency (MMRD) hypermutated tumours [11]. Exposure to ionising radiation is to be avoided in patients with Li-Fraumeni or Gorlin syndrome and chromosomal breakage syndromes to prevent subsequent cancers. Conventional doses of myelosuppressive chemotherapy (specifically alkylating agents) with the attendant excess toxicity should be avoided in chromosomal breakage syndromes and those with defective DNA repair. Understanding of CPS helps in planning organ-sparing surgery (e.g., nephron-sparing surgery in Wilms tumour), risk-reducing surgery (e.g., prophylactic thyroidectomy in MEN2 syndrome) and in donor selection for allogenic stem cell transplantation. In rhabdomyosarcoma (RMS), significantly worse outcomes were demonstrated with germline TP53 or HRAS alterations, especially for fusion-negative RMS, suggesting a possible role of these for future risk stratification [12].

A study by Villani *et al*, [13] showed that a comprehensive surveillance protocol for individuals with pathogenic TP53 variants improved long-term survival, facilitating this approach in these patients [13]. Patients with constitutional MMRD may also benefit from cancer surveillance, especially for colorectal cancer and brain tumours, though surveillance may not guarantee detection at a curable stage [14]. Recommendations for surveillance and position statements in most key CPS have recently been published, based on expert opinion, though the impact of surveillance on survival is not established [15].

### Cancer predisposition syndromes: Indian perspective

The expanding field of germline genetic testing is still in its nascent stage in LMICs, including India. Lack of awareness among patients and primary health-care providers, restricted access to germline genetic testing services, compounded by cost and limited availability of genetic counsellors, are hindrances to the implementation of these services. Moving ahead, the phenotypic spectra, frequency, genotype-phenotype correlations and geo-ethnic variations are hitherto unexplored. This necessitates a comprehensive germline genetic testing in consecutive patients in a research context to assess the prevalence and impact of a CPS diagnosis on treatment adaptation, survival outcomes, quality of life, economic resources and psychosocial outcomes. These baseline data could inform further testing policies and help develop an algorithm that could be integrated into routine clinical practice. Phenotype-driven, curated gene panels appear to be more cost-effective and practical in contemporary settings [10]. Incorporation of genetic services into the national cancer control programme would be a desirable step in the long term.

In India, the establishment of dedicated pediatric genetics clinics in tertiary care centers have been initiated [16]. The institution of a Cancer Genetics Subcommittee under the Indian Pediatric Hematology Oncology Group (INPHOG), a leading collaborative network in the country, is a welcome step toward streamlining the services [17]. Multi-center studies involving major institutes in the country are ongoing, and leverage the existing infrastructure, expertise and data-sharing frameworks. These studies are anticipated to further facilitate the development of nodal centers offering laboratory, online genetic counselling and other support in a hub and spoke model.

## Immunotherapy

Immunotherapy has emerged as a ground-breaking approach in the treatment of various pediatric cancers, leveraging the body’s immune system to destroy cancer cells. The use of immunotherapy may potentially reduce long-term toxicity – a critical consideration in pediatric oncology. Additionally, immunotherapies are uniquely suited to achieving remission or positive responses in relapsed/refractory cancers that have proven resistant to traditional treatments like chemotherapy and radiation.

### Immunotherapy: current evidence

#### Antibody-based immunotherapy

*Unconjugated monoclonal antibodies* attach directly to the target cell surface antigens, causing cell lysis. The prototype, rituximab, has now evolved as the standard of care in the management of high-risk mature B-NHL in children [18]. It is also widely used in salvage regimens in Hodgkin and other CD20-positive NHLs.

*Antibody drug conjugates (ADCs)* are coupled to cytotoxic drugs, targeting specific cell surface antigens and delivering the cytotoxic payload to the target cells. ADCs useful in pediatric cancers include Inotuzumab, Gemtuzumab (humanised anti-CD33-calicheamicin conjugate), Brentuximab [anti-CD30 monoclonal antibody-monomethyl auristatin E conjugate] for leukemia/lymphoma [19, 21]. Inotuzumab has shown remarkable efficacy in CD22-positive relapsed/refractory adult and pediatric acute lymphoblastic leukemia (ALL) and is being investigated in the frontline setting in childhood ALL. Gemtuzumab significantly reduced relapse risk in childhood acute myeloid leukemia in the Children’s Oncology Group (COG) AAML0531 study [20]. Similarly, Brentuximab has shown a remarkable response in CD30-expressing lymphomas (Hodgkin lymphoma, ALCL) and is currently projected as a standard treatment for children with high-risk Hodgkin lymphoma [21].

*Bispecific antibodies* are engineered antibodies recruiting immune cells to the close proximity of the cancer cells for effecting cytotoxicity. Blinatumomab (anti-CD19) is the most important molecule in this group and is now approved for the treatment of relapsed/refractory ALL in both children and adults [22]. Based on recent reports from the COG-AALL1731, children treated with blinatumomab in combination with chemotherapy had improved outcomes over chemotherapy alone in non-high-risk B-ALL in the frontline setting [23]. Blinatumomab has also shown exceptional efficacy in infant ALL and other subsets of very high-risk pre B-cell ALL.

The most prominent example of *monoclonal antibodies developed for solid tumours* is dinutuximab, targeting the GD2 antigen expressed on neuroblastoma cells. A reported 20% survival benefit over conventional therapy in high-risk neuroblastoma patients led to its approval in this setting [24]. A high response rate with chemoimmunotherapy is also demonstrated in the relapse/refractory setting [25]. Humanised or chimeric anti-GD2 are being explored in other GD2+ pediatric solid tumours, including osteosarcoma and soft tissue sarcomas.

Despite their efficacy, antibody-based therapies are not without limitations. Infusion reactions, cytokine release and neurotoxicity are common adverse effects [26]. Additionally, resistance can develop due to antigen loss or downregulation, necessitating combination therapies or sequential treatments [27].

#### Cell-based immunotherapy

Cell-based immunotherapy focuses on the use of modified immune cells to target and destroy cancer cells. Approval of tisagenlecleucel (Kymriah), an anti-CD19 chimeric antigen receptor (CAR) T-cell for pediatric and young adult patients with relapsed/refractory B-ALL, marked a significant milestone [28]. Clinical trials have reported remission rates exceeding 80%, even in heavily pre-treated populations [29]. CAR-Ts against other leukemia antigens such as CD-22 (B-cell ALL), CD7 (T-ALL/AML), CD5 (T-ALL) and CD123 (AML) are under various stages of development and clinical trials as is newer generations of CAR-T constructs aimed at increased specificity and efficacy with minimal toxicity.

CAR T-cell therapies are also being explored in solid tumours including relapsed/refractory neuroblastoma, though with less success [30]. The challenges include the heterogeneity of solid tumour antigens, the immunosuppressive tumour microenvironment and physical barriers preventing T-cell infiltration.

#### Immune checkpoint inhibitors

These block proteins are used by cancer cells to evade immune detection, thereby reactivating T-cells to attack the tumour. The most studied ICI target programmed cell death protein 1 (PD-1), its ligand PD-L1 and cytotoxic T-lymphocyte-associated protein 4. Their role in pediatric cancers is still emerging [31]. Pediatric tumours generally have a lower mutation burden, resulting in fewer neoantigens and reduced immunogenicity compared to adult tumours. However, certain pediatric cancers, particularly those with DNA repair deficiencies or microsatellite instability, may respond well to checkpoint blockade [11]. Among ICI, pembrolizumab and nivolumab have been extensively studied in pediatric cancers like relapsed/refractory Hodgkin lymphoma, high-grade gliomas with mismatch repair deficiency or high microsatellite instability 11,26 [11]. Despite their promise, ICI in children carries potential risks, including autoimmune toxicities like colitis, endocrinopathies and pneumonitis. Long-term follow-up is essential to monitor these effects and better understand the impact of immune modulation on developing organs. Table 1 lists the main immunotherapeutic agents approved for use in pediatric cancers.

## Immunotherapy: Indian perspective

Significant challenges remain in the universal application of immunotherapy, ranging from toxicity management to the high cost of therapies [32, 33]. Furthermore, limited availability makes these therapies inaccessible to most children from the LMICs. Infrastructure deficiencies, including a lack of specialised facilities and trained personnel, hinder proper administration. Ongoing research and clinical trials are expected to expand the availability and effectiveness of these treatments [32]. Regulatory barriers and logistical issues, such as a lack of approvals/slow approvals and cold-chain requirements, further delay access. Financial constraints, including limited funding and prohibitive out-of-pocket costs, exacerbate disparities. Also, underrepresentation in clinical trials leads to a lack of region-specific data, compounded by ethical and logistical difficulties in conducting research in all ethnicities. Addressing these issues requires targeted investments, policy reforms, advent of generic formulations, humanitarian/compassionate access to these high-end therapies [33].

In India, the development of an indigenous CAR-T cell therapy is a promising development, with NexCAR19 approved for adolescents and young adults [34]. In children, this has successfully completed a phase 1 trial and is now progressing to phase 2 studies. Furthermore, blinatumomab is being offered to children at 7 hospitals in 4 LMICs through a humanitarian access program, organised by Amgen in collaboration with St. Jude Children’s Research Hospital in Memphis, USA [35]. Other therapeutic agents, such as Inotuzumab, are also marketed in India and are being used in clinical practice to treat children with ALL.

## Precision medicine

Over the last decade, precision medicine has significantly advanced in oncology, offering tailored treatment approaches based on the molecular and genetic characteristics of tumours. While still in its incipient stage in pediatric oncology, early data underscores its potential, particularly for high-risk and rare cancers with poor outcomes [27, 28].

### Precision medicine: current evidence

Studies such as the NCI-COG Pediatric MATCH trial have laid the foundation for the broader application of precision medicine in pediatric oncology [36]. This trial established a collaborative framework for efficiently collecting, processing and sequencing refractory pediatric cancers. Early results revealed actionable alterations in 31.5% of the first 1,000 tumours screened. Similarly, the European MoleculAr profiling for pediatric and young adult cancer treatment stratification (MAPPYACTS) trial identified potentially actionable targets in 69% of 624 patients screened [37]. Beyond identifying actionable mutations, these studies have unveiled other critical benefits. Germline mutations were detected in 7%–17% of enrolled patients, offering insights into hereditary cancer predisposition with significant implications for family members [38]. Molecular profiling has also occasionally led to changes in diagnosis, enhancing the precision of therapeutic strategies [39]. Once actionable targets are identified, patients can be enrolled in platform trials targeting specific survival pathways or have their cases reviewed in multidisciplinary tumour boards (MTBs). MTBs provide age-specific therapeutic recommendations, ensuring safety and efficacy for pediatric patients. Table 2 lists the Published pediatric precision medicine studies.

Despite progress, challenges persist. While actionable mutations are identified in a significant proportion of cases, only a fraction of patients receive matched targeted therapies. For instance, in the MAPPYACTS trial, only 30% of patients with actionable mutations accessed targeted treatments [37]. Barriers include the unavailability of drugs within clinical trials and uncertainties surrounding efficacy and benefit-risk balance. Another recurring concern is the outcomes of targeted therapies resulting from a precision medicine-based approach. Preliminary results from the NCI-MATCH study showed limited responses for agents such as palbociclib (CDK4/6 inhibitor), ulixertinib (ERK1/2 inhibitor) and samotolisib (PI3K/mTOR inhibitor) [40–42]. However, encouraging results have been seen in specific high-risk or relapsed cancers with key driver mutations such as *NTRK* fusions, *ALK* mutations and *BRAF* mutations, as shown in the MAPPYACTS and INFORM registry [37]. Based on these results, agents are now being explored in frontline settings. However, a notable gap in existing studies is the lack of comprehensive survival outcome data, with most focusing on objective response rates. To unlock the full potential of precision medicine, long-term outcomes must be prioritised in future research.

### Precision medicine: Indian perspective

Implementing precision medicine in LMICs presents unique challenges. Access to advanced diagnostic tools like whole-genome sequencing, whole-exome sequencing and methylation studies is limited, necessitating adaptive and alternative strategies (for example, use of clinicoradiological and histological characteristics to make a molecular diagnosis in medulloblastoma) [43]. Another concern in LMIC is the availability and affordability of corresponding targeted therapies for actionable mutations. Unlike conventional chemotherapy, most targeted therapies are non-myelosuppressive, which significantly improves the quality of life for patients and their caregivers, due to the decreased incident morbidities. Continuous efforts need to be made to enrich and update the WHO essential medicine list for children to increase access to more targeted therapies. Standardised reporting of treatment responses and survival outcomes is crucial to assess the true impact of precision medicine in LMICs. National MTBs can play a critical role in LMICs by providing a platform to discuss molecular profiling results and therapeutic options for complex cases. Based on current evidence, even in LMICs, precision-based approaches should initially be restricted to clinical trials focusing on high-risk pediatric cancers with survival rates below 30%. Establishing funded clinical trials with centralised testing and collaborative frameworks is essential, which can ensure equitable access to precision medicine, even in resource-constrained settings, while generating robust data to guide treatment strategies. There is a need for strategic investments in research infrastructure, international collaborations and clinical trial frameworks.

In India, initiatives such as virtual tumour board organised by the National Cancer Grid, have enabled clinicians to incorporate reports of molecular testing in the risk stratification and treatment algorithms, and to promote the judicious resource-adapted use of NGS and molecular testing, providing a framework for future molecular tumour boards [44]. Government initiatives, like the Department of Health Research’s collaboration with the Indian Council of Medical Research under the DIAMOnDS project, have established guidelines for advanced molecular oncology diagnostics, ensuring wider implementation of these technologies [45]. The main objective of the project is to strengthen the diagnostic research and geographical spread of services and provide free-of-cost diagnostic services and evidence-based health care for patients with cancer. Enhancing access to targeted therapies by implementing patient assistance and compassionate-use programs, along with promoting the availability of cost-effective generic formulations, will significantly advance equitable and affordable care.

## Survivorship

The flipside of improved long-term survival is the increased risk of a wide spectrum of life-altering and life-threatening late effects in a proportion of childhood cancer survivors (CCSs) contributing to morbidity and mortality [3]. A report from the childhood cancer survivor study (CCSS) cohort identified that two out of three survivors will develop a chronic health condition, and more than one third will develop a condition that is severe or life-threatening [46]. Acknowledging the need for evidence-based, lifelong surveillance of health-related outcomes, the COG and subsequently, the International Late Effects of Childhood Cancer Guideline Harmonisation Group (IGHG) have developed risk-based exposure-related guidelines for follow-up care of CCSs [47, 48].

### Survivorship: current evidence

As childhood cancer remains a rare disease, recognition of low prevalence, yet clinically relevant late effects, poses a challenge due to the low event rate. Multiple institutions and cooperative groups have built and followed up cohorts of CCSs [49–51]. Combining data from various cohorts could make way for early identification of late effects, both known and novel, and updating of surveillance recommendations in long-term follow-up clinical practice guidelines. A recent pooled data analysis of more than 25,000 survivors from the CCSS, the Dutch Children’s Oncology Group’s later study and the St Jude lifetime study demonstrated the dose equivalence among anthracyclines with regards to cardiomyopathy, thereby impacting the screening recommendations for recipients of anthracycline with/without chest radiotherapy [52, 53]. Moving forward, similar collaborations facilitated in predicting the late effect risks of specific therapeutic exposures such as development and validation of a risk-prediction model for heart failure, ischemic heart disease and stroke, prediction of risk for acute ovarian failure and calculation of polygenic risk scoring in assessing the risk for developing subsequent thyroid cancer in CCSs [54–56]. Recognition of specific exposure-related late effects initiated risk-adapted treatment approaches, thereby modifying the side-effect profile. Details in Table 3. The contributions of genetic factors/pharmacogenomics in the genesis of late effects in CCSs, especially in health conditions like SMN, cardiovascular disease, obesity and hearing loss remains elusive till date. Multinational collaborative efforts with clinical, laboratory inputs in these domains would expedite research across a diverse patient profile and foster the development of preventive as well as frontline treatment strategies [57]. Longitudinal studies in the survivors of recipients of novel treatments with survival benefit and varied late-effect profiles like targeted therapy, immunotherapy, cellular therapy and proton beam radiotherapy are the need of the hour. A recent study reported on the long-term toxicity profile of CAR-T cell therapy [58]. One of the evolving areas in cancer survivorship research is the focus on chronic health conditions in young adult survivors, leading to frailty, which can lead to early mortality. With the increase in the proportion of adult survivors in various cooperative groups, the focus is on identifying risk factors for frailty and determining the effectiveness of pharmacologic and lifestyle interventions in preventing or delaying the premature aging process [59]. The complexity of survivorship care, with the continued emergence of new knowledge, poses a challenge in its implementation. One important way used in bridging the gap between the knowledge and practice is developing and timely updating the survivorship guidelines. The IGHG is developing a living guidelines information and communication technology (ICT) tool in collaboration with a European project (PanCareFollowUp). This ICT tool, ‘the living guideline’, will be able to regularly search the literature and provide the guideline groups with new evidence. It will also support the guideline groups to grade new evidence and, if needed, adjust current recommendations. This will be instrumental in translating new knowledge into guidelines in an efficient way [60].

### Survivorship: Indian perspective

Accessibility, diversity in care models and barriers to childhood cancer survivorship care is a well-known, unique factors determining the delivery of quality care to the survivors [61]. In HICs, most CCSs have access to long-term follow-up (LTFU) care by their primary oncology team or a dedicated LTFU clinic. Research suggests that survivors who attend LTFU care programs have better long-term physical and psychosocial health outcomes than non-attenders [62]. Replicating similar models in LMICs is fraught with challenges [63]. Implementation barriers can relate to survivor, health-care provider and health-care domains. Survivors’ issues include knowledge deficits, lack of endorsement of need for LTFU care by the treating physician, perception that LTFU was not necessary because they felt well and were not experiencing problems, and competing demands related to school, work or family [64]. Health-care provider issues consist of knowledge deficits, lack of experience with survivorship care, especially with management of survivors with multisystem morbidity, lack of resources, lower priority for survivorship versus acute oncology care and suboptimal communication among providers [61, 65]. To optimise the delivery of CCS care, concerted efforts are required to enhance knowledge in patient and family about the need and potential benefits of LTFU care, improve provider education and training, and bring in policy change to ensure that childhood cancer survivors have access to essential services and resources to improve their quality of life. Importantly, there needs to be a focus on pre-empting the development of late toxicities by using up-to-date risk-adapted protocols, monitoring for toxicities on treatment and improving capacity for procedures such as fertility preservation [64].

India’s first dedicated multidisciplinary survivorship clinic was established at Tata Memorial Hospital, Mumbai, which has registered more than 5,500 CCS till date. A report published by this group identified the spectrum of late effects prevalent in their cohort of survivors, reflective of the issues more commonly described in LMICs [63]. There is a promising future with the implementation of innovative steps to improve survivorship care in India with a focus on decentralisation of care, shared-care and remote follow-up models for low-risk survivors, increased participation from non-profit organisations, incorporation of technology in generating individualised survivorship-care plans, telesurvivorship and collaborative research within the Late Effects subcommittee of INPHOG and Indian childhood cancer initiative [64, 65].

## Summary

In the contemporary era, a paradigm shift in pediatric oncology care is evident across multiple domains, including cancer predisposition, immunotherapy, precision medicine and survivorship, designed to harmonise survival with quality of life, ensuring the best possible outcomes. Though the above changes are increasingly evident in HICs, several hurdles and challenges exist in the implementation of these strategies in LMICs. Substantial changes are requisite to facilitate the translation of these changing paradigms into reality in LMICs.

## List of abbreviations

LMIC, Low and middle-income countries; HIC, High-income countries; CPS, Cancer predisposition syndrome; NGS, Next generation sequencing; MMRD, Mis match repair deficiency; ALL, Acute lymphoblastic leukemia; AML, Acute myeloid leukemia; ADC, Antibody drug conjugate; COG, Children’s oncology group; CAR T, Chimeric antigen receptor T; MTB, Multidisciplinary tumour boards; CCS, Childhood cancer survivor; LTFU, Long term follow up; IGHG, International Late Effects of Childhood Cancer Guideline Harmonisation Group; ICI, Immune checkpoint inhibitors; INPHOG, Indian Pediatric Hematology Oncology Group.

## Conflicts of interest

The authors declare that there is no conflicts of interest.

## Funding

Nil funding received.

## Author contributions

Dr Badira Cheriyalinkal Parambil conceptualised and designed the article, drafted the initial manuscript and reviewed and revised the manuscript. Drs Nirmalya Roy Moulik, Venkata Rama Mohan Gollamudi, Shyam Srinivasan, drafted the initial manuscript and reviewed and revised the manuscript. Drs Maya Prasad and Girish Chinnaswamy conceptualised and designed the article, and reviewed and revised the manuscript. Drs Gaurav Narula, Chetan Dhamne, Akanksha Chichra, Mukta Ramadwar, Poonam Panjwani, Siddhartha Laskar, Nehal Khanna, Jifmi Jose Manjali, Sajid Qureshi, Vasundhara Patil, Akshay Baheti, Sneha Shah, Kunal Gala, P G Subramanian, Prashant Tembhare, Nikhil Patkar, Gaurav Chatterjee, Swetha Rajpal, Dhanlaxmi Shetty, critically reviewed the manuscript for important intellectual content and revised the same. All authors have contributed in significant ways, have reviewed and agreed upon the manuscript content.

## Figures and Tables

**Table 1.** Immunotherapeutic agents approved for use in pediatric cancers.

Agent	Disease indicated	Year of approval	Key studies/findings	Availability in India
Pembrolizumab (anti-PD-1)	Hodgkin lymphoma (relapsed/refractory), tumors with MSI-H or dMMR	2020 (MSI-H/dMMR approval for pediatrics)	Shown to improve outcomes in relapsed/refractory Hodgkin lymphoma and MSI-H/dMMR tumors like high-grade gliomas. Key trials: keynote series.	Marketed in India
Nivolumab (anti-PD-1)	Hodgkin lymphoma (relapsed/refractory)	2020 (pediatrics)	Demonstrated high response rates and durable remissions in pediatric Hodgkin lymphoma. Studies indicate safety and efficacy in younger populations.	Marketed in India
Blinatumomab (BsAb)	B-cell acute lymphoblastic leukemia (relapsed/refractory)	2016 (pediatrics)	Showed significant improvement in minimal residual disease (MRD)-negative remission rates. Major trial: tower.	Not marketed, access through humanitarian/compassionate access program, or imported against payment on a named patient basis
Dinutuximab (anti-GD2)	High-risk neuroblastoma	2015	Improved survival in combination with GM-CSF, IL-2, and isotretinoin. Key trial: COG-ANBL0032.	Not marketed, imported against payment on a named patient basis
Tisagenlecleucel (CAR T)	B-cell acute lymphoblastic leukemia (relapsed/refractory)	2017	First CAR T-cell therapy approved for pediatric ALL. Achieved high remission rates in the ELIANA trial.	Not marketed, imported against payment on a named patient basis. Phase 2 study on children of an indigenous product currently ongoing.
Gemtuzumab ozogamicin (ADC)	Acute myeloid leukemia (CD33-positive)	2017	Demonstrated efficacy in pediatric AML in combination with chemotherapy. Key trial: AAML0531 showed improved event-free survival in younger patients.	Not marketed, imported against payment on a named patient basis.

**Table 2.** Published pediatric precision medicine studies (38).

Trial	Tumors included	Number of samples analysed	Percentage of actionable alterations (%)	Patients receiving targeted therapy (%)	Response to targeted therapy
Mappycats	Relapsed/refractory Solid and CNS tumors; hematological malignancies	632	56	19	Objective response rate:17% Stable disease: 25%
Ped-match	Relapsed/refractory Solid and CNS tumors; hematological malignancies	1,000	32	13	Reported separately in different treatment agonistic studies
Inform	Primary high-risk; Relapsed/refractory Solid and CNS tumors; hematological malignancies	926	48	16	Superior PFS among patients with very high priority level target
Kics	Poor prognosis; rare tumors; cancer predisposition. Solid and CNS tumors; hematological malignancies	252 264	54	15	NA
Zero childhood cancer program	Primary high-risk; relapse/refractory. Solid and CNS tumors; hematological malignancies	252	53	17	Objective response: 31%
SMPaeds	Relapse/refractory solid tumors	255	51	2	NA
iTHER	Primary high-risk; relapse/refractory cancers Solid and CNS tumors; hematological malignancies	106	44	14	Complete remission: 5%

PFS- Progression free survival, CNS-Central nervous system

**Table 3.** Impact of risk-adapted treatment in pediatric oncology on late effects profile in CCSs (66–68).

First author, Year	Disease	Change in management based on risk stratification	Impact
**Hodgson [66]**	Hodgkin lymphoma	Decrease in volume/field of RT: From extended field RT to IFRT and INRT Decrease in RT dose Response-adapted selection of patients for RT	Decrease in incidence of SMN (up to 20-fold higher risk on historic treatments using extended field RT) Decrease in cardiac morbidity (up to 2-4-fold increased risk on receiving 35–45 Gy RT)
**Friedman *et al* [67]**	Neuroblastoma	Risk-adapted treatment	SMR _high_ =27.7 (21.4–35.8) SMR _intermediate_ =3.3 (1.7–6.5) SMR _low_ =2.8 (1.7–4.8) Decrease in SMR in low and intermediate-risk SMN risk: SIR_ high_ =28.0 (8.5–42.3) SIR _intermediate_ =3.7 (1.2–11.3) No increased SMN risk in low-risk Grade 3–5 Chronic health conditions: HR _high_ =16.1 (11.2–23.2) HR _intermediate_ =6.3 (3.8–10.5) HR _low_ =1.8 (1.1–3.1) Decreased HR in in low and intermediate-risk
**Essig *et al* [68]**	Acute lymphoblastic leukemia- standard risk	Less intense chemotherapy regimens Omission of cranial RT	Only 1% survivors developed SMN (SIR-2·6, 95%CI:1·0–5·7) No significant impact on educational attainment, rate of marriage, or independent living.

RT-Radiotherapy, IFRT-Involved field RT, INRT-Involved node RT, SMN- Subsequent malignant neoplasms, SMR-Standardised mortality ratio, SIR-Standardised incidence ratio, HR-Hazard ratio.
